# Research activity amongst DCM research priorities

**DOI:** 10.1007/s00701-021-04767-6

**Published:** 2021-02-24

**Authors:** Ben Grodzinski, Harry Bestwick, Faheem Bhatti, Rory Durham, Maaz Khan, Celine Iswarya Partha Sarathi, Jye Quan Teh, Oliver Mowforth, Benjamin Davies

**Affiliations:** 1 School of Clinical Medicine, University of Cambridge, Cambridge, UK; 2 Academic Neurosurgery Unit, Department of Clinical Neurosurgery, University of Cambridge, Cambridge, UK

**Keywords:** Cervical, Myelopathy, Spondylosis, Spondylotic, Degeneration, Degenerative

## Abstract

**Introduction:**

Degenerative cervical myelopathy (DCM) is a progressive neurodegenerative disorder. DCM is common (estimated prevalence, 2% of adults) and significantly impacts quality of life. The AO Spine RECODE-DCM (*Re*search Objectives and *Co*mmon *D*ata *E*lements in DCM) project has recently established the top research priorities for DCM. This article examines the extent to which existing research activity aligns with the established research priorities.

**Methods:**

A systematic review of MEDLINE and Embase for “Cervical” AND “Myelopathy” was conducted following PRISMA guidelines. Full-text papers in English, exclusively studying DCM, published between January 1, 1995 and August 08, 2020 were considered eligible. Extracted data for each study included authors, journal, year of publication, location, sample size and study design. Each study was then analysed for alignment to the established research priorities.

**Results:**

In total, 2261 papers with a total of 1,323,979 patients were included. Japan published more papers (625) than any other country. Moreover, 2005 (89%) of 2261 papers were aligned to at least one research priority. The alignment of papers to the different research priorities was unequal, with 1060 papers on the most researched priority alone (#15, predictors of outcome after treatment), but only 64 total papers on the least-researched 10 priorities. The comparative growth of research in the different priorities was also unequal, with some priorities growing and others plateauing over the past 5 years.

**Discussion:**

Research activity in DCM continues to grow, and the focus of this research remains on surgery. The established research priorities therefore represent a new direction for the field.

**Supplementary Information:**

The online version contains supplementary material available at 10.1007/s00701-021-04767-6.

## Introduction

Degenerative cervical myelopathy (DCM) is a progressive neurological condition defined by symptomatic spinal cord compression caused by arthritic narrowing of the spinal canal [4]. It is common, estimated to affect as many as 2% of adults [10], and has a significant impact on health-related quality of life [9].

The AO Spine RECODE-DCM (*Re*search Objectives and *Co*mmon *D*ata *E*lements in DCM) project is an international collaboration, supported by the AO Spine Foundation and Myelopathy.org, which aims to accelerate knowledge discovery in DCM, through a number of consensus initiatives, including a James Lind Alliance priority setting process to establish the top research priorities for DCM (Table 1). Research priorities were selected through consensus from a longlist of 74 unanswered research questions. The longlist was formed by first consolidating 3404 research suggestions, from an international and multi-disciplinary community into unique research questions, and second checking current evidence to ensure these are unanswered (aospine.org/recode).

**Table 1:** Top 26 priorities for DCM research. Priorities are listed in descending order of established importance. Each priority has a unique single-letter ID, a ‘key phrase’ and a set of associated questions

Priority #	Question	Key phrase
1	What strategies can increase awareness and understanding of DCM amongst healthcare professionals and the public? Can these strategies help improve timely diagnosis and management of DCM?	Raising awareness
2	What is the natural history of DCM? What is the relationship between DCM and asymptomatic spinal cord compression or canal stenosis? What factors influence the natural history of the disease?	Natural history
3	What are the diagnostic criteria of DCM? When should imaging be used in the assessment of DCM?	Diagnostic criteria
4	How can the severity of DCM be evaluated? What assessment tools can be used to evaluate functional impairment, disability and quality of life in people with DCM? What instruments, tools or methods can be used or developed to monitor people with DCM for disease progression or improvement either before or after surgical treatment? Is there a role for smart technology?	Assessment and monitoring
5	What is the pathophysiology of DCM? What are the mechanisms of neurological injury and the molecular and anatomical consequences?	Pathophysiology
6	What is the role of non-operative or peri-operative management or rehabilitation for DCM? What are the most effective strategies?	Rehabilitation
7	Can novel therapies, including stem-cell, gene, pharmacological and neuroprotective therapies, improve the health and wellbeing of people living with DCM and slow down disease progression?	Novel therapies
8	What is the socio-economic impact of DCM (the financial impact of living with DCM to the individual, their supporters and society as a whole)?	Socio-economic impact
9	What is the role of dynamic imaging and novel, unconventional or advanced techniques in the assessment of DCM?	Imaging techniques
10	Are there clinical and imaging factors that can help a surgeon select who should undergo surgical decompression in the setting of DCM? At what stage of the disease is surgery the preferred management strategy?	Individualising/staging of surgery
11	What are the main signs and symptoms that people with DCM present with? What are the frequency, sensitivity, specificity and positive predictive value of symptoms and signs (clinical assessments) for DCM?	Signs and symptoms
12	What is the optimal follow-up for people with DCM managed conservatively and surgically? What is the appropriate follow-up for people with DCM or those with spinal cord compression but no myelopathy? Who should follow these individuals? How often should new imaging be obtained? How should changes in neurological status be documented or addressed?	Follow-up
13	What are the most effective therapies for treatment of specific symptoms of DCM and the prevention of associated complications in DCM, including spasticity, imbalance and sensory, bladder or bowel dysfunction?	Symptom management
14	What are the factors that predict the development of myelopathy in people with evidence of spinal cord compression and no symptoms?	Predictors of development
15	What are the most important determinants of functional outcomes, quality of life and patient satisfaction following surgical or non-operative treatment for DCM?	Predictors of outcome, QoL, and satisfaction
16	What clinical and/or imaging features are predictive of neurological deterioration in people with DCM? Are there certain features that indicate irreversible disease?	Predictors of progression
17	What are the risk factors for the development or progression of DCM, including but not limited to lifestyle, diet, exercise, posture, occupation, history of trauma and coexistent disease? Does their modification have a role in prevention or treatment?	Risk factors for development/progression
18	What is the ideal timing for surgical intervention?	Timing of surgery
19	What is the efficacy and safety of non-operative treatment in the management of DCM compared with surgical treatment? Can non-operative treatment avoid the need for surgery long-term? When can a ‘watch and wait’ approach be adopted?	Non-operative treatment
20	What are the most effective therapies for treating pain in people with DCM?	Pain management
21	What is the preferred management strategy for people with mild DCM? What is the most cost-effective management strategy in this cohort? Are there clinical and imaging features that predict who should undergo surgical decompression and/or when?	Management of mild DCM
22	Can cerebrospinal fluid (CSF) or serum biomarkers be identified to support early diagnosis of DCM, and/or predict treatment outcomes?	Biomarkers
23	What lifestyle modifications (such as physical activity or exercise) are required or should be recommended to people with DCM to support recovery, avoid deterioration and improve quality of life?	Lifestyle modifications
24	What is the role of surgery in the management of people with imaging evidence of cord compression but no specific features of myelopathy? Is this decision impacted by signal change on T2-weighted MRI images or the presence of neck pain?	Surgery for pre-myelopathic compression
25	What treatments should be implemented following surgery and continued in the long-term? Is there a role for extended rehabilitation and exercise programmes? What should be its frequency, content and duration, and whom should it be coordinated by?	Post-operative treatment
26	What is the incidence of adjacent segment degeneration following surgery for DCM? Are there strategies that can reduce the incidence of adjacent segment degeneration?	Adjacent segment degeneration

In order for this prioritisation process to have its desired effect, the priorities must be adopted by the research community. Therefore, a next stage of the project involves promoting their uptake. This process, known often as knowledge translation (KT), is critical in bridging the gap between the acquisition of knowledge (in this case, the research priorities) and the implementation of this knowledge into practice (ensuring that future research is aligned with the priorities).

The first step in the KT process is to establish a baseline to define the gap that needs to be bridged and a reference point for which KT interventions can be measured against. This article therefore aims to establish the extent to which existing and emerging research activity aligns with the newly identified research priorities.

## Methods

Primary clinical trials contained within the Myelopathy.org Literature Database on DCM were evaluated for alignment with the research priorities included in the final face-to-face consensus meeting.

### Myelopathy.org literature database

Systematic review and meta-analysis, otherwise known as research synthesis, are important tools to quantitatively summarise current knowledge about a topic. Their findings are fundamentally underpinned on the studies that they identify. Within the field of DCM, there is inconsistent disease terminology, with an absence of index terms or codes [7], which makes literature searching extremely inefficient. For this reason, and to support work by the Myelopathy.org charity, a hand indexed database of primary clinical articles has been maintained.

The dataset was built using a search of Embase and MEDLINE for [“Cervical”] AND [“Myelopathy”]. It includes primary clinical human trials, in which DCM is the primary condition being addressed, with the full text available in English. Animal studies, case reports, letters, editorials, reviews, technical notes, commentaries, proposals and corrections were excluded.

The database was initially built on previous systematic reviews [2, 3] and initially contained all papers published up to December 31, 2015 [8]. The last update was performed on August 08, 2020. This updated database forms the basis of this study.

### Analysis

Each paper was assessed (by authors BG, HB, FB, RD, MK, CIPS, JQT) for alignment to the established research priorities. Each paper was allocated to one or more priorities, or deemed to be not aligned to any priority.

The following information was extracted for each paper: title, abstract, author names, country of corresponding author, year of publication, research theme (as per [8]), patient characteristics (number of patients, surgical vs. non-surgical treatment) and study design (prospective or retrospective, cohort or case-control, level of evidence).

Statistical analysis was used to detect differences in research activity between research priorities and over time. SPSS Statistics (IBM Corporation, Armonk, NY) was used for all calculations. Shapiro-Wilk tests were used to analyse normality of data, and parametric or non-parametric tests were used accordingly. Significance was set at *p* < .05. We report mean ± standard deviation unless otherwise specified.

## Results

### Summary of global dataset

**Fig. 1:**
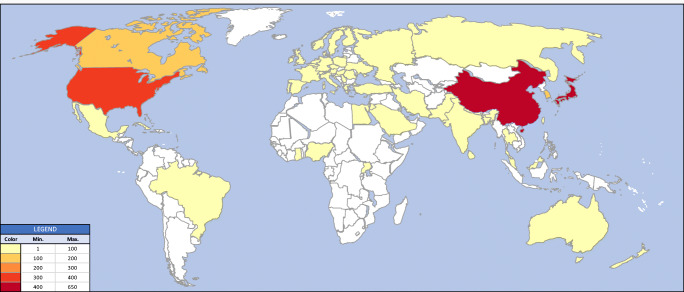
Global distribution of DCM research activity. Country colour indicates number of papers published in that country, as per the legend. The country attributed is that of the lead author

In total, 2261 papers, with a total of 1,323,979 patients (median, 63 patients in the 2230 articles that reported an *N)*, were included. Japan has published more papers (625, 27.6% of total) than any other country, whilst the USA studied the greatest number of patients (4,549,916). The aggregate global distribution of research activity is shown in Fig. 1 and per priority in Supplementary Data 3. Moreover, 727 (32.2%) papers reported a prospective design, whilst 806 (35.6%) studies reported a retrospective design. Furthermore, 728 (32.2%) papers had an unreported/unclear design.

### Aggregate data per priority

Summary data for each priority are shown in Table 2. A substantial proportion (256/2261, 11.3%) of papers were not aligned to any priority. Those papers assigned to a priority were unevenly distributed. For instance, 1060/2261 (47%) of papers were assigned to priority #15 (predictors of outcome after treatment), whilst there were nine priorities that had fewer than 10 papers each (Table 3).

**Table 2:** Aggregate data for each priority

Priority #	N	Mean	Median	Std. deviation	Minimum	Maximum	% of total *N*
/	249	429.91	60.00	3656.988	4	55,346	11.2%
1	7	88.86	89.00	64.955	8	187	0.3%
2	45	23,191.29	127.00	111,044.621	4	714,654	2.0%
3	26	81.08	58.50	64.974	12	244	1.2%
4	298	1269.14	66.00	18,700.061	3	322,869	13.4%
5	66	79.62	48.50	105.231	5	743	3.0%
6	8	6848.25	49.00	19,220.342	9	54,416	0.4%
7	26	90.38	42.50	92.078	5	280	1.2%
8	18	5212.39	187.50	12,602.700	36	50,605	0.8%
9	178	1501.21	63.00	10,118.974	5	109,728	8.0%
10	49	4162.00	88.00	20,637.732	6	141,450	2.2%
11	33	5969.91	109.00	33,041.216	4	190,021	1.5%
12	13	751.31	54.00	2500.028	3	9071	0.6%
13	3	47.67	36.00	34.990	20	87	0.1%
14	12	75.75	65.00	45.965	17	156	0.5%
15	1046	2519.87	64.50	42,632.284	2	1,323,979	46.9%
16	21	2810.43	42.00	12,490.841	8	57,323	0.9%
17	29	87.86	44.00	168.768	7	916	1.3%
18	7	273.14	208.00	235.099	110	778	0.3%
19	15	1139.80	79.00	4046.535	14	15,761	0.7%
20	9	38.78	39.00	19.156	8	73	0.4%
21	3	69.00	47.00	40.731	44	116	0.1%
22	35	130.37	45.00	208.829	1	1009	1.6%
23	3	46,530.33	587.00	79,901.167	212	138,792	0.1%
24	4	77.00	67.50	63.577	11	162	0.2%
25	7	127.71	45.00	170.041	8	481	0.3%
26	20	269.70	54.50	708.927	12	3209	0.9%
Total	2230	2346.73	63.00	34,630.011	1	1,323,979	100.0%

*N* number of papers, *Mean* mean number of patients per paper, *Median* median number of patients per paper. *Std. Deviation* is the standard deviation of *N* within each priority. *Maximum* and *minimum* refer to number of patients in each paper. *% of total N* shows the percentage of all patients in each priority. The total number of papers shown here (2230) is less than total number of papers in the database (2261) because not all papers have an N (i.e. not all papers reported the number of patients)

**Table 3:** Number of papers per priority. This table shows all papers in this study, including those that did not report an *N* (unlike Table 2)

Priority #	Frequency	Percent
/	256	11.3
1	8	.4
2	45	2.0
3	26	1.1
4	299	13.2
5	67	3.0
6	8	.4
7	26	1.1
8	18	.8
9	181	8.0
10	49	2.2
11	34	1.5
12	13	.6
13	3	.1
14	12	.5
15	1060	46.9
16	21	.9
17	30	1.3
18	7	.3
19	15	.7
20	9	.4
21	3	.1
22	37	1.6
23	3	.1
24	4	.2
25	7	.3
26	20	.9
Total	2261	100.0

**Fig. 2:**
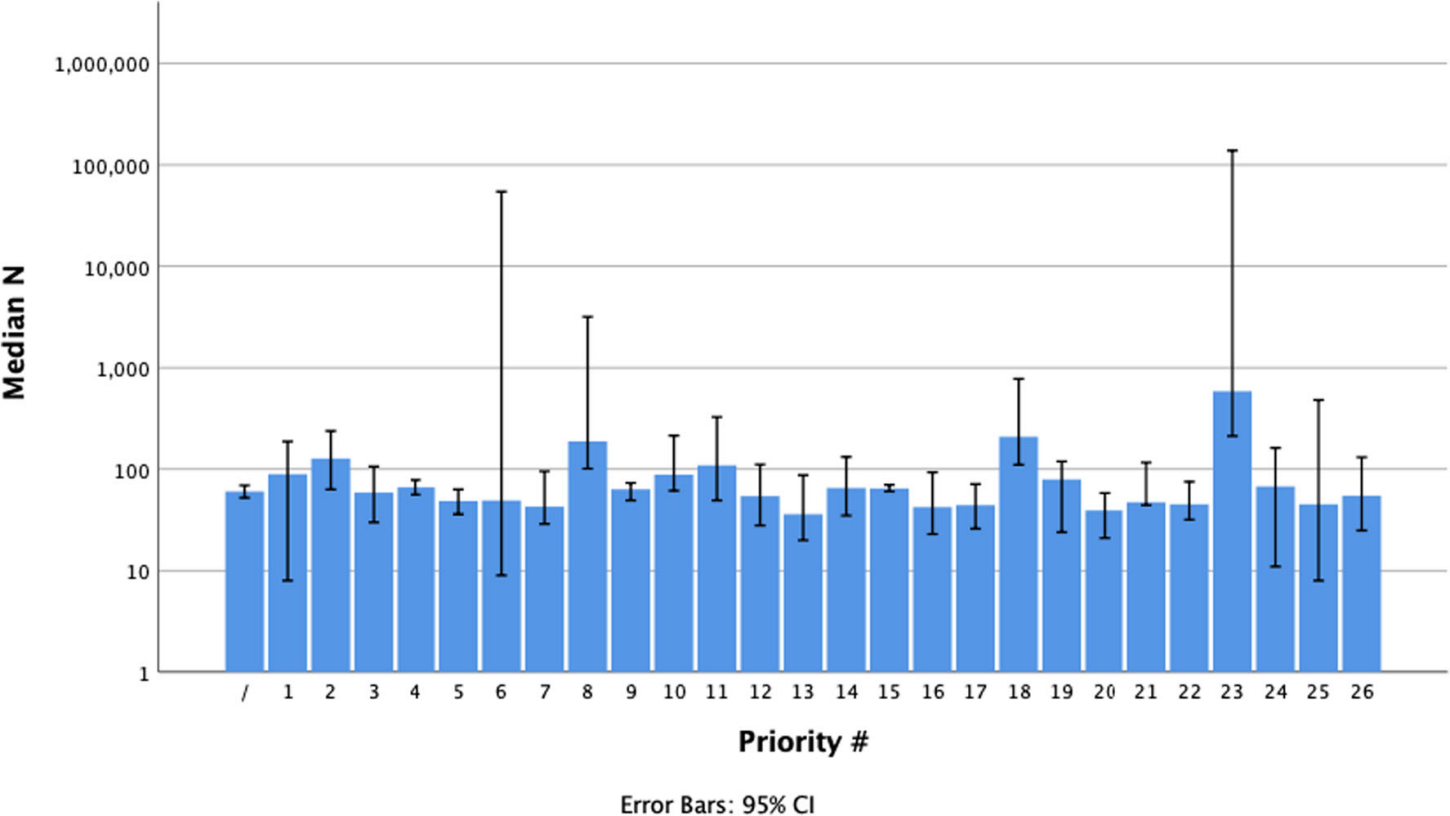
Median paper sample size by priority. Note the logarithmic scale on the vertical axis. Error bars indicate 95% confidence intervals

Median sample sizes for each priority are shown in Fig. 2. There was a statistically significant difference in the distribution of sample sizes between priorities (*p* = <0.001, independent-samples Kruskal-Wallis test).

### Research trends

**Fig. 3:**
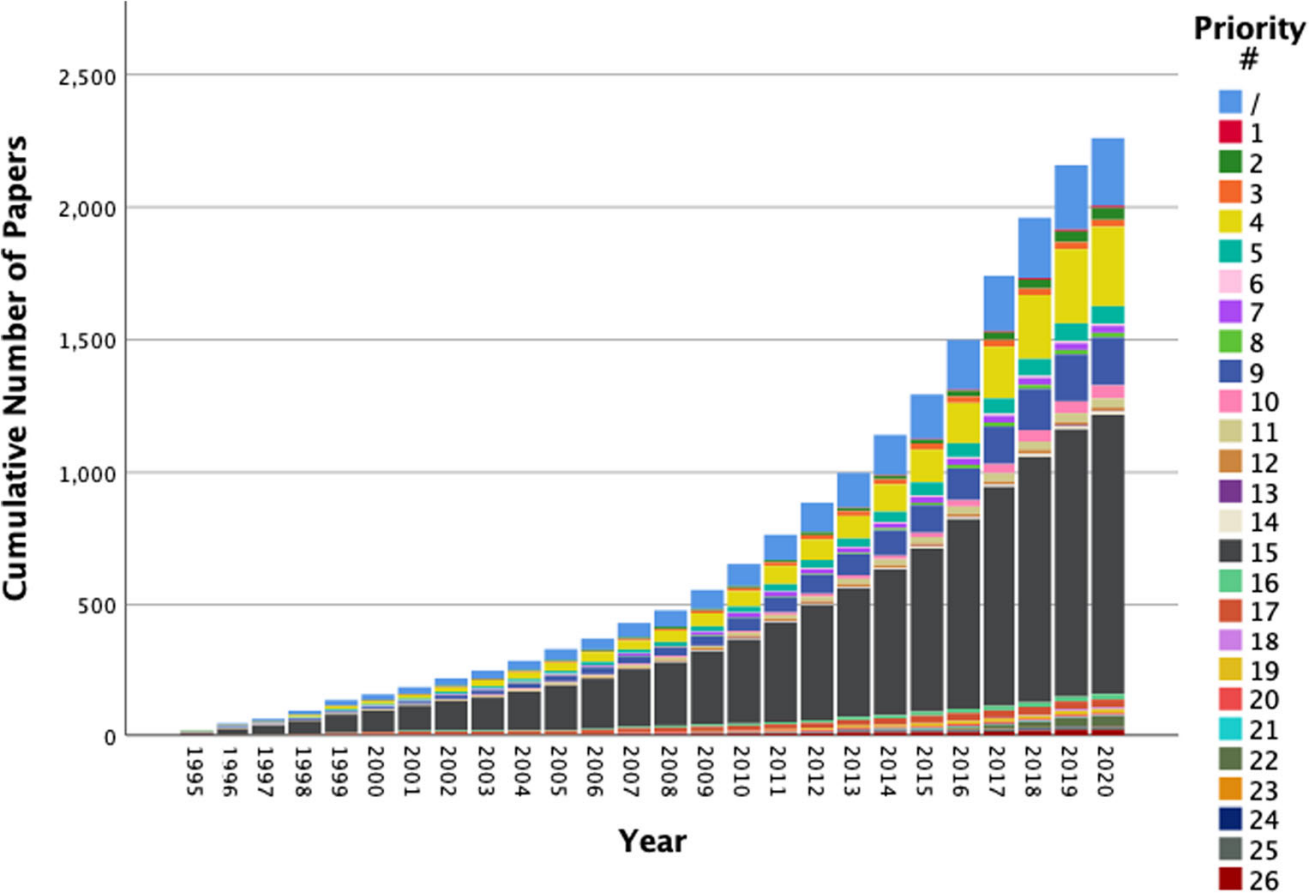
Cumulative number of papers by priority. Cumulative counts for each priority are shown in different colours. Note that the data for 2020 is up until February 12, and hence, the expected end-of-year counts will likely be higher

The research activity in DCM over time is shown in Fig. 3 and Supplementary Data 2. Growth of research activity in the different priorities is significantly different (*p* = <0.001, Pearson Chi-squared test). For example, one priority (#21, management of mild DCM) had no papers published in the past 5 years, whilst another priority (#13, symptom management) had 33% of all its papers published in the past 5 years. Within the last 5 years, three priorities have shown consistent growth – #4 (assessment and monitoring), #9 (imaging techniques) and #15 (predictors of outcome after treatment).

## Discussion

Research activity in DCM continues to grow, and whilst a number of priorities appear to be an increasing focus for researchers, the majority of published research is not aligned with the newly established research priorities with many priorities showing little aligned activity.

### Research activity in DCM continues to grow

This study was based on 2261 papers that form the entire literature in DCM over the past 25 years – a relatively small research output compared to other diseases of similar prevalence. Despite this, research activity has continued to grow since our last evaluation [8], with 968 new papers since January 1, 2016. In line with our previous findings, the focus of DCM research remains on surgery. In particular, the determinants of outcome after surgery (aligned with Priority #15) have been a particular focus. These determinants may include the choice and timing of the surgical procedure [6, 11, 12].

### Research activity in DCM does not align with newly established research priorities

With the exception of Priority #15 (determinants of post-treatment outcome), the established research priorities have been under-represented. We found more papers not aligned to any research priority (‘/’, 256 papers) than aligned to any individual priority other than #15 and #4 (Table 3). This is emphasised when examining the most important priorities – the #1 priority (raising awareness) has only eight papers aligned to it, and the top ten priorities have only 780 papers between them, less than 35% of the 2261 included papers. Furthermore, there is inequality in research activity amongst the research priorities – both in cumulative counts (Tables 2 and 3) and in recent growth (Fig. 3).

It should be acknowledged that these priorities are newly established and reflect on-going clinical uncertainties. The literature included in this review spans 25 years, during which time the research priorities may have been different. For example, it is notable that during this period the evidence base for surgical management has increased significantly (as reflected within the AO Spine Guidelines [5]), which is in line with substantial research activity [8]. These findings therefore do not dismiss previous research but aim to highlight the present directions and knowledge translation gap. However, this example does highlight the logical correlation between a research focus and clinical progress, substantiating within DCM the premise and potential for research priority setting [1].

### Role of this paper

This paper therefore serves several roles. Firstly, it acts as a collection of research papers, comprising the entire current DCM literature, to serve as a reference for current research. Secondly, it indicates the magnitude of change required to address the research priorities, indicating which priorities in particular are under-researched. Both the established order of importance and the existing number of papers aligned to each priority may be used to guide researchers in choosing a topic for research. Thirdly, it acts as a baseline, against which the success of the AO Spine RECODE-DCM project, and in particular its KT strategy, may be measured against in the future.

### Limitations

As we have published previously [7], owing to the difficulties in indexing DCM literature, the results presented here are based upon an extensive hand search of the literature (title and abstract only). Whilst this may introduce an element of subjectivity – the reviewers must decide which papers meet the pre-established criteria and which do not and this is not always clear – this was the preferred approach to ensure a more comprehensive representation of the field. Given this breadth and the number of studies captured, we are confident that the overall findings are a true reflection of the DCM research field, although we note the need for reproducibility of methods in future research of this type. Establishing a clear set of disease codes to combat this problem is one of the aims of the AO Spine RECODE-DCM project and will help to address this for the future.

## Conclusion

Previous and emerging DCM research does not align with the newly established research priorities. This poses a challenge for the uptake and implementation of the research priorities. However, the strong correlation between surgical research activity over the last 25 years and the advances for its evidence indicate that if this can be achieved it will pay dividends.

## Supplementary information

ESM 1 (DOCX 5184 kb).

## Declarations

### Disclaimer

This report is an independent research arising from a Clinical Doctoral Research Fellowship supported by the National Institute for Health Research. The views expressed in this publication are those of the authors and not necessarily those of the NHS, the National Institute for Health Research or the Department of Health and Social Care.
