# Supplementary material for: Research activity amongst DCM research priorities
Source: Acta Neurochir (Wien). 2021 Feb 24;163(6):1561–8. doi: 10.1007/s00701-021-04767-6 (PMC8116279; doi:10.1007/s00701-021-04767-6)
Supplement: Supplementary file 1 — (DOCX 5184 kb). [file 701_2021_4767_MOESM1_ESM.docx]

# Supplementary Files

## Supplementary Data 1

Supplementary Data 1. **Figure:** **Paper occurrences over time for each priority**. Yearly counts for each priority are shown in different colours. Note that the data for 2020 is up until August 08, and hence the expected end-of-year counts will likely be higher.

## Supplementary Data 2


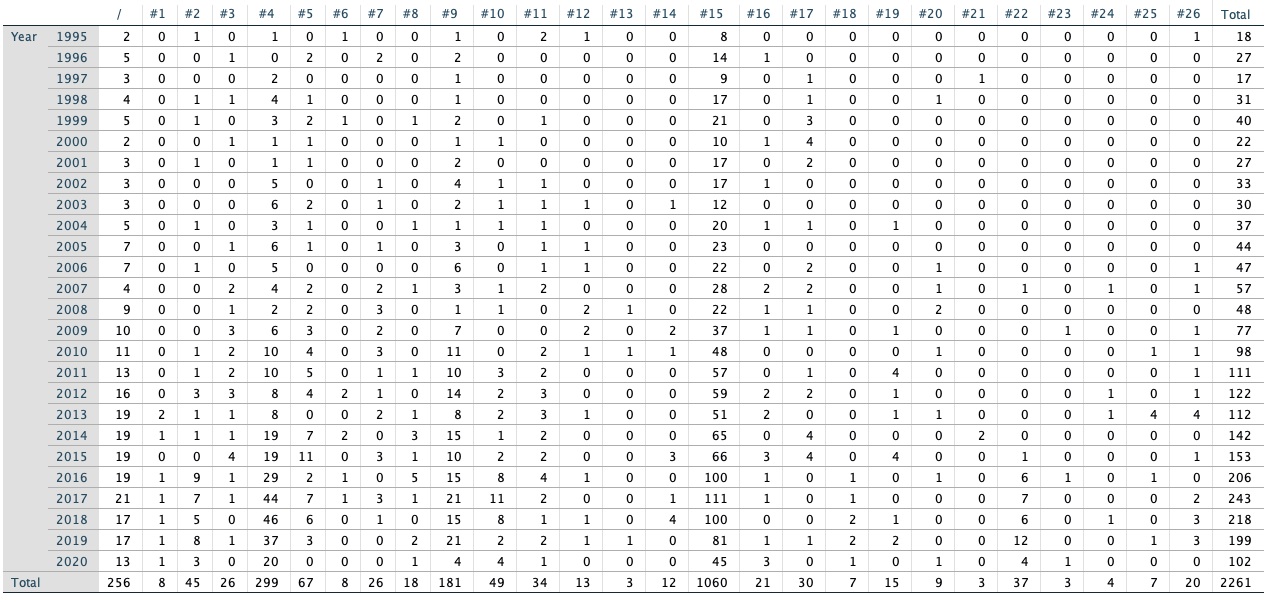


Supplementary Data 2. **Table:** **Paper occurrences over time for each priority.** Yearly counts for each priority are shown. Note that the data for 2020 is up until August 08, and hence the expected end-of-year counts will likely be higher.

## Supplementary Data 3

### Not aligned to any priority

### Priority 1

### Priority 2

### Priority 3

### Priority 4

### Priority 5

Supplementary Data 3. **Global distribution of DCM research activity by priority**. Distributions for the top 5 priorities, as well as papers assigned to no priority, are shown. As in Figure 1, country colour indicates number of papers published in that country. The country attributed is that of the lead author.
